# Comparison of two diagnostic strategies for patients with stable chest pain suggestive of chronic coronary syndrome: rationale and design of the double-blind, pragmatic, randomized and controlled OPERATE Trial

**DOI:** 10.1186/s12872-023-03424-3

**Published:** 2023-08-23

**Authors:** Jia Zhou, Ting Xin, Yahang Tan, Jianzhong Pang, Tao Chen, Hao Wang, Jia Zhao, Chang Liu, Cun Xie, Minghui Wang, Chengjian Wang, Yuanying Liu, Jie Zhang, Yankun Liu, Chen Shanfu, Chunjie Li, Hongliang Cong

**Affiliations:** 1 Clinical School of Thoracic, Tianjin Medical University, Tianjin, China; 2 Department of Cardiology, Tianjin Chest Hospital, Tianjin, China; 3 Department of Cardiology, Tianjin First Central Hospital, Tianjin, China; 4 Department of Cardiology, Beijing Chaoyang Hospital, Capital Medical University, Beijing, China; 5 Department of Cardiology, Tianjin Second Teaching Hospital of Tianjin University of Traditional Chinese, Tianjin, China; 6 Department of Emergency, Hebei Petrochina Central Hospital, Langfang, Hebei China; 7 Department of Clinical Epidemiology and Evidence-Based Medicine, Friendship Hospital, Capital Medical University, Beijing, China

**Keywords:** Diagnostic strategy, Cardiac imaging testing, Stable chest pain, Chronic coronary syndrome, Randomized controlled trial, Coronary computed tomography angiography, Major adverse cardiovascular event, Pretest probability

## Abstract

**Background:**

To achieve potential financial savings and avoid exposing the patients to unnecessary risk, an optimal diagnostic strategy to identify low risk individual who may derive minimal benefit from further cardiac imaging testing (CIT) is important for patients with stable chest pain (SCP) suggestive of chronic coronary syndrome (CCS). Although several diagnostic strategies have been recommended by the most recent guidelines, few randomized controlled trials (RCTs) have prospectively investigated the actual effect of applying these strategies in clinical practice.

**Methods:**

OPERATE (**OP**timal **E**valuation of stable chest pain to **R**educe unnecess**A**ry utilization of cardiac imaging **TE**sting) trial is an investigator-initiated, multicenter, coronary computed tomography angiography (CCTA)-based, 2-arm parallel-group, double-blind, pragmatic and confirmative RCT planning to include 800 subjects with SCP suggestive of CCS. After enrollment, all subjects will be randomized to two arms (2016 U.K. National Institute of Health and Care Excellence guideline-determined and 2019 European Society of Cardiology guideline-determined diagnostic strategy) on a 1:1 basis. According to each strategy, CCTA should be referred and deferred for a subject in high and low risk group, respectively. The primary (effectiveness) endpoint is CCTA without obstructive coronary artery disease. Safety of each strategy will be mainly assessed by 1-year major adverse cardiovascular event rates.

**Discussion:**

The OPERATE trial will provide comparative effectiveness and safety evidences for two different diagnostic strategies for patients with SCP suggestive of CCS, with the intension of improving the diagnostic yield of CCTA at no expense of safety.

**Clinical trial registration:**

ClinicalTrial.org Identifier NCT05640752.

**Supplementary Information:**

The online version contains supplementary material available at 10.1186/s12872-023-03424-3.

## Background

In daily clinical routine, the evaluation of new-onset stable chest pain (SCP) suggestive of chronic coronary syndrome (CCS) remains a challenge for physicians, in consideration of the decreasing prevalence of coronary artery disease (CAD) and dramatically rising costs related to these patients [1, 2]. Although coronary computed tomography angiography (CCTA) has been the first-line cardiac imaging testing (CIT) according to current recommendations [3–5], an increasing body of evidence pointed to the fact that most of patients referred to CCTA as well as other CIT had normal results and no adverse clinical events [6–10]. Thus, an optimal diagnostic strategy to identify low risk patients who may derive minimal benefit from further CIT is the cornerstone of clinical management for SCP, which has been proposed and reiterated by most recent guidelines to achieve potential financial savings and avoid exposing patients to unnecessary risk [3–5].

Different diagnostic strategies have been developed to defer unnecessary CIT. The 2016 U.K. National Institute of Health and Care Excellence (NICE) guideline abandoned pretest probability (PTP)-based strategy and recommended the referrals of CCTA in patients with angina or abnormal ECG [5]. On the contrary, the 2019 European Society of Cardiology (ESC) guideline suggested an updated PTP model to rule out CAD in patients with ESC-PTP ≤ 5% and recommended noninvasive CIT for patients with ESC-PTP ≥ 5% [3]. For patients with borderline ESC-PTP (5–15%), Winther et al. developed the risk factor-weighted clinical likelihood (RF-CL) model, which was also proven to improve the prediction of CAD [11]. Both ESC-PTP and RF-CL model were recommended by the latest guideline for evaluation and diagnosis of chest pain [4].

2016 NICE guideline-determined diagnostic strategy (NICE strategy) [12, 13] and ESC guideline-determined diagnostic strategy (ESC strategy) [11, 14, 15] were externally validated and compared in different CCTA-based cohorts of SCP patients, as well as our previous studies from the CCTA Improves Clinical Management of Stable Chest Pain (CICM-SCP) registry [16–18]. While evidences from these observational cohorts and post hoc analyses of randomized controlled trial (RCT) suggested that the PTP-based strategy offered more effective deferral for CIT than the symptom-focused strategy did [16], they only examined patients who had underwent CIT and indications for CIT relied on a nonrandomized and complicated fashion, resulting in substantial biases. In fact, few RCTs have prospectively determined the actual effect of applying these strategies in clinical practice [19, 20].

Therefore, the OPERATE (**OP**timal **E**valuation of stable chest pain to **R**educe unnecess**A**ry utilization of cardiac imaging **TE**sting) study is designed to compare the effectiveness and safety of two proposed diagnostic strategies, ESC and NICE strategy, in identification of low risk individual who may derive minimal benefit from CCTA among patients with SCP suggestive of CCS in a pragmatic RCT.

## Methods/design

### Overall design and objectives

**Fig. 1:**
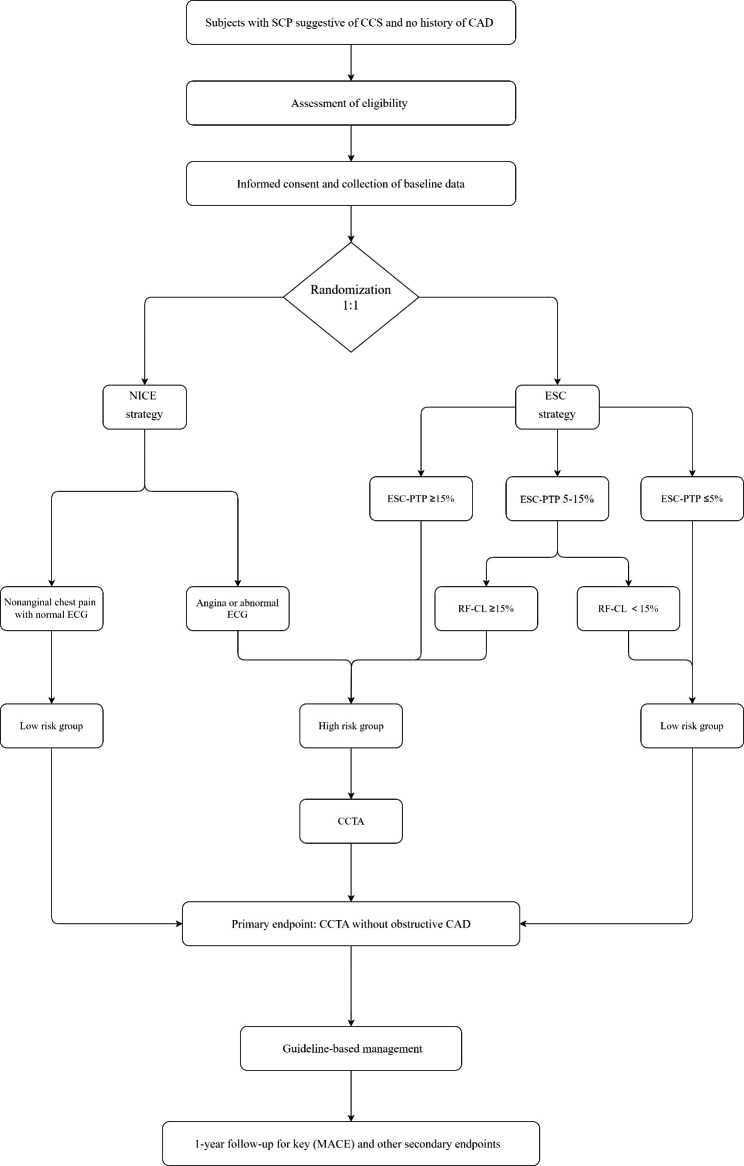
Study design SCP: stable chest pain; CCS: chronic coronary syndrome; CAD: coronary artery disease; ECG: electrocardiogram; NICE strategy: 2016 National Institute of Health and Care Excellence guideline-determined strategy; ESC strategy: 2019 European Society of Cardiology guideline-determined strategy; MACE: major adverse cardiovascular event; CCTA: coronary computed tomography angiography; RF-CL: risk factor-weighted clinical likelihood; PTP: pretest probability

Figure 1 shows an overview of the study design. OPERATE trial is an investigator-initiated, multicenter, CCTA-based, 2-arm 1:1 parallel-group, double-blind, pragmatic and confirmative RCT planning to include 800 subjects with SCP suggestive of CCS (ClinicalTrials.gov Identifier NCT05640752). This study protocol is developed in accordance with the Standard Protocol Items: Recommendations for Interventional Trials (SPIRIT) [21] and the SPIRIT checklist is available in Additional file 1. The primary objective of OPERATE trial is to compare the rates of CCTA without obstructive CAD according to NICE and ESC strategy. The key secondary objective is to assess whether the two strategies have no significant difference in terms of major adverse cardiac events (MACEs). We hypothesize that when comparing with NICE strategy, ESC strategy which sequentially incorporated the ESC-PTP model with RF-CL model will decrease the probability of CCTA without obstructive CAD but not at the expense of safety and cost over a follow-up period of 1 year.

### Enrollment and screening

Subjects will be enrolled competitively from five major cardiac centers recognized as tertiary A level (Tianjin Chest Hospital, Tianjin First Central Hospital, Beijing Chaoyang Hospital, Tianjin Second Teaching Hospital of Tianjin University of Traditional Chinese and Hebei Petrochina Central Hospital) in Beijing-Tianjin-Hebei region, which has more than 100 million population. The total annual number of patients presenting with SCP suggestive of CCS and referred to CIT in these high-volume sites is more than 30,000 and 20,000, respectively. Subjects are considered eligible for inclusion if they suffer SCP suggestive of CCS, are more than 30 years of age, and willing and able to give informed consent. Eligible subjects are clinically stable, have no history of CAD and will undergo routine electrocardiogram (ECG) and echocardiography according to the latest guideline [3, 4]. Trained research nurses introduce the trial to subjects and investigators are advised not to randomize subjects who expressed a clear preference for undergoing CIT or not during the informed consent process. More details about inclusion and exclusion criteria are listed in Table 1.

**Table 1:** Inclusion and exclusion criteria

Inclusion criteria	1. SCP or equivalent^a^ suggestive of CCS and clinically stability
	2. No history of CAD (prior myocardial infarction, CR or any CAD documented by previous CIT)
	3. Age ≥ 30 years
	4. Willing and able to provide informed consent
Exclusion criteria	1. Prior CIT within 1 year prior to randomization
	2. Clinically instability (e.g. cardiogenic shock, ACS, severe arrhythmias or NYHA III or IV heart failure)
	3. Non-sinus rhythm
	4. Concomitant participation in another clinical trial
	5. Complex structural heart disease
	6. Non-cardiac illness with life expectancy < 2 years
	7. Allergy to iodinated contrast agent
	8. Estimated glomerular filtration rate < 60 ml/min/1.73m^2^ within 90 days
	9. Body mass index > 35 kg/m^2^
	10. Expressing a clear preference for undergoing CIT or not
	11. Pregnancy

CIT: cardiac imaging testing; CCS: chronic coronary syndrome; SCP: stable chest pain; CAD: coronary artery disease; ECG: electrocardiogram; NCCT: non-contrast CT; ACS: acute coronary syndrome; CR: coronary revascularization

a: The followings are not SCP:

1) First appearance within the last 48 h or Canadian Cardiovascular Society Angina Grading Scale (CCSAGS) Class IV

2) Progressive with at least 1 CCSAGS Class to at least CCSAGS Class IV

3) Now at rest for at least 30 min

### Baseline clinical data

Baseline characteristics are defined and collected as described previously [16, 18, 22]. Hypertension is defined as blood pressure of ≥ 140/90 mmHg or the use of anti-hypertension medication. Hyperlipidemia is defined as total cholesterol of ≥ 220 mg/dL, low-density lipoprotein cholesterol of ≥ 140 mg/dL, fasting triglycerides of ≥ 150 mm/dL or receiving treatment with oral lipid-lowering agents. Diabetes is defined as fasting glucose levels over 7 mmol/L or treatment currently with either diet, oral glucose lowering agents or insulin. Smoking is defined as current smoking or smoking in past 6 months. Abnormal ECG is defined pathological Q waves or ST-segment and T wave abnormalities in at least two adjacent leads. A family history of premature CAD is defined as diagnosis of the disease in a male first-degree relative before 55 years of age or in a female first-degree relative before 65 years of age. Typical angina is defined as having 3 characteristics: (1) substernal discomfort of characteristic quality, (2) precipitated by physical exertion or emotion, and (3) relieved with rest or nitroglycerin within 10 min. Atypical angina is defined as having 2 of the 3 definition characteristics. Nonanginal chest pain is characterized as chest pain or discomfort that meets 1 or 0 of the 3 definition characteristics [23].

### Randomization and blinding

After collection of baseline clinical data, eligible subjects will be randomized (in blocks of 2) sequentially 1:1 to start double-blind diagnostic strategy with 1 of 2 regimens: NICE and ESC. The computerized randomization list is generated independently by a statistician from the statistical and data coordinating group who will not participate rest of the study. Both investigators and subjects are blinded to the allocation process until the end of trial (sequentially numbered, opaque, sealed envelope will be used to conceal allocation). Once the subject is ready for randomization, the corresponding envelope will be opened by the independent strategy assignment expert panel consisting of cardiologists who will not participate rest of the study. The expert panel determine whether the subject should be referred to CCTA according to the given diagnostic strategy (see below) and only send the final recommendation about referral or deferral of CCTA to the physician and subject.

### Diagnostic strategies

For a subject in high risk group according to each strategy, CCTA should be referred as the first-line CIT and other CIT, including noninvasive functional testing (NFT) and invasive coronary angiography (ICA), will be considered as an alternative. In both diagnostic strategies, subjects determined to be at low risk will be referred to optimal medication treatment (OMT) with no immediate CIT. The decisions regarding OMT will be done at discretion of the referring physicians according to recent primary prevention guidelines [24, 25], and is not a part of the study protocol. Thus, in cases where symptoms don’t resolve with maximum OMT, additional cardiac or non-cardiac diagnostic testing could be pursued as an escalation of the diagnostic strategy. Based on current SCP guidelines [3–5] and results of proposed researches [11–17], details of risk groups are illustrated in Fig. 1; Table 2 and as follows:

**Table 2:** Low and high risk groups according to NICE and ESC strategy

	NICE strategy	ESC strategy
Low risk group	Both normal ECG and nonanginal chest pain	ESC-PTP ≤ 5%, or both 5%< ESC-PTP < 15% and RF-CL < 15%
High risk group	Typical and atypical angina, or nonanginal chest pain with abnormal ECG	ESC-PTP ≥ 15%, or both 5%< ESC-PTP < 15% and RF-CL ≥ 15%

ECG: electrocardiogram; NICE strategy: 2016 National Institute of Health and Care Excellence guideline-determined strategy; ESC strategy: 2019 European Society of Cardiology guideline-determined strategy; RF-CL: risk factor-weighted clinical likelihood; PTP: pretest probability

According to NICE strategy, subjects with nonanginal chest pain and normal ECG are classified into low risk group. Subjects with typical and atypical angina or nonanginal chest pain with abnormal ECG are classified into high risk group [5].

For each subject, ESC-PTP is calculated using age, sex and type of chest pain according to 2019 ESC guideline for the diagnosis and management of CCS [3] and RF-CL is calculated using age, sex, type of chest pain, hypertension, dyslipidemia, diabetes, smoking and family history of CAD based on the publication of Winther et al. [11], respectively. According to ESC strategy, subjects with ESC-PTP ≤ 5% are classified into low risk group and ones with ESC-PTP ≥ 15% are classified into high risk group [3]. For subjects with ESC-PTP of 5-15%, ones with RF-CL ≥ 15% are classified into high risk group and ones with RF-CL < 15% are classified into low risk group. The cut-off of 15% for RF-CL is chosen because RF-CL < 15% was associated with an extremely low prevalence of obstructive CAD and risk of clinical events [11, 16].

### CCTA and other CIT

All participating sites will use standard equipment, procedure and interpretation for CIT, as defined by current practice guidelines [3, 4, 26–29] and local protocols as previously described in the CICM-SCP registry [16, 22, 30]. All results will be reported by independent radiology/cardiology consultants with a minimum 5-year experience in the imaging modality and provided to the local physicians for subsequent decision making. The site interpretation of CIT was verified by central review of the clinical reports with review of the primary images as needed [31, 32]. The exercise electrocardiography criterion for a positive test was greater ≥ 1.5 mm of horizontal or down sloping ST segment deviation (depression or elevation) in any 2 leads except aVR for at least 60 to 80 ms after the end of the QRS complex, either during or after exercise. The criterion of single-photon emission computed tomography or positron emission tomography for a positive test was area of ischaemia ≥ 10% of the left ventricle myocardium. The stress echocardiography criterion for a positive test was ≥ 3 of 16 segments with stress-induced hypokinesia or akinesia. The criterion of cardiac magnetic resonance for a positive test was ≥ 2 of 16 segments with stress perfusion defects or ≥ 3 dobutamine-induced dysfunctional segments. The fractional flow reserve (FFR) derived from CCTA criterion for a positive test was FFR < 0.8.

Specially, CCTA images are acquired using 64-slice, or greater, multidetector CT scanners and all DICOM imaging data will be sent to the CCTA Core Laboratory (Tianjin Chest Hospital) for further analysis. In the CCTA Core Laboratory, all CCTA images will be analyzed by three experienced observers, 2 radiologists and 1 cardiologist, who are blinded to the other data. In image analyses, all segments ≥ 2 mm in diameter are identified and analyzed using the updated Coronary Artery Disease–Reporting and Data System (CAD-RADS) [29]. The percent diameter stenosis in every segment is categorized as 0% (no stenosis), 1–24% (minimal stenosis), 25–49% (mild stenosis), 50–69% (moderate stenosis), 70–99% (severe stenosis), and 100% (total occlusion). Interobserver disagreements are resolved by consensus.

The result of CCTA, as well as other CIT, will be provided to the local physician and he or she will make subsequent clinical decisions for the subject, such as additional CIT and clinical interventions like cardiac rehabilitation, OMT and coronary revascularization (CR), based on recommendations from clinical guidelines [3, 4, 26–29] and his or her cumulative clinical assessment of the subject.

### Study endpoint

The primary outcome, main secondary outcomes and other outcomes are described below and all outcomes are included Table 3. The primary endpoint of the study which indicates effectiveness of the diagnostic strategy is CCTA without obstructive CAD, defined as the summary of nonobstructive CAD, no sign of CAD and nondiagnostic result detected by CCTA according to each strategy during initial management. Obstructive CAD is defined as present if a patient had at least one lesion with ≥ 50% diameter stenosis at CCTA.

**Table 3:** Study endpoints

Outcome	Topic	Description
Primary	CCTA without obstructive CAD	Summary of nonobstructive CAD, no sign of CAD and nondiagnostic result on CCTA during initial management
Secondary		
Safety	MACE	Composite of death, nonfatal MI and hospitalization due to unstable angina
	Individual cardiovascular endpoint	All-cause death, nonfatal MI and hospitalization due to unstable angina, respectively
	Exposure to radiation	Cumulative estimated radiation exposure related to cardiovascular care during the whole study period
	Procedural complications	Major: renal failure, stroke, severe bleeding and anaphylaxis or other complications if they lead to a hospital stay of at least 24 h Minor: complications not leading to prolonged stay, such as slight decrease of renal function, anaphylaxis and bleeding
Effectiveness	No. of cardiovascular procedures	Noninvasive CIT: exercise ECG, CCTA, PET-CT, MRI, myocardial perfusion imaging and stress echocardiography Invasive procedures: ICA and CR (CABG and PCI)
	Diagnostic metrics of CCTA during initial management	
	Diagnostic yield	No. of obstructive CAD/No. of CCTA×100%
	Proportion of necessary CCTA	No. of necessary CCTA/No. of CCTA×100%
	Proportion of normal CCTA	No. of normal CCTA/No. of CCTA×100%
	Other outcomes	HRQOL: Seattle Angina Questionnaire and visual-analogue scale of the European Quality of Life–5 Dimensions Time length of initial management: from successful eligibility to the end of initial management

CIT: cardiac imaging testing; MI: myocardial infarction; ECG: electrocardiogram; CR: coronary revascularization; HRQOL: health-related quality of life; MACE: major adverse cardiovascular events; CCTA: coronary computed tomography angiography; PET-CT: positron emission tomography-CT; MRI: magnetic resonance imaging; ICA: invasive coronary angiography; CABG: coronary artery bypass grafting; PCI: percutaneous coronary intervention

The key secondary endpoint indicating the safety of diagnostic strategy is the time to first MACE using composite of the following clinical events: All-cause death is used rather than cardiovascular death to eliminate the need for possibly difficult adjudication of causes of death, especially given the relatively low mortality; Nonfatal myocardial infarction (MI), including spontaneous and procedure-related, is defined according to the Fourth Universal Definition of Myocardial Infarction [33]; Hospitalization due to unstable angina is defined as an event in which the final diagnosis is myocardial ischemia (at least one of the following must be present: dynamic ST depression, ischemia on stress testing or significant epicardial coronary artery stenosis) and either of the following criteria is present: ischemic discomfort or equivalent symptoms requiring hospitalization within 48 h of symptoms and lasting at least 10 min at rest, or occurring in an accelerated pattern within 48 h of hospitalization. Only symptomatic events are defined as MACE. Asymptomatic events, such as silent MI is defined as incidental findings–if they will be detected at all.

In addition to the individual components of MACE, other secondary endpoints indicating the safety of diagnostic strategy include the followings: All-cause death, nonfatal MI, hospitalization due to unstable angina, overall exposure to radiation and complications (classified as major and minor) related to cardiovascular procedures which occur within 72 h. The estimated cumulative radiation exposure over the entire trial will be measured in milliSieverts using administered dose on CCTA, converted contrast agent dose for nuclear and administered radiation dose (kerma air product or dose length product) or fluoroscopy time for angiography [34].

Another secondary endpoint indicating the effectiveness of diagnostic strategy is resource use patterns (cumulative number of cardiovascular procedures during both initial management and follow-up, such as noninvasive and invasive CIT or CR). The diagnostic metrics of CCTA during initial management, including diagnostic yield proportion of normal CCTA and necessary CCTA, will be also calculated. Necessary CCTA is defined as obstructive CAD or alteration of OMT due to nonobstructive CAD on CCTA. Other effectiveness outcomes include time length of initial management and health-related quality of life (HRQOL).

### Study period and follow-up

The planned duration of the study is 24 months: 12 months for the enrollment period, defined as first visit of first subject to first visit of last subject and 12 months for the follow-up period. Study will preliminarily end when the following have occurred: at least 800 subjects have been randomized and 12 ± 1 month have elapsed since the last subject is randomized. All subjects will be followed for a minimum of 1 year and we will also try to continue the further follow-up after 1 year at 12-month intervals until death or withdrawal. Follow-up will be performed at 1st month (± 7 days), 3rd moth (± 14 days), 6th month (± 14 days), and 12th month (± 28 days) after randomization. The information of clinical visits is shown in Table 4. For outcome evaluation and recording of test complications, subjects have either a telephone call or clinic visit and the medical records are collected by trained interviewers. Data collected for suspected events are provided to the independent clinical event adjudication committee masked to the strategy assignment and intervention.

**Table 4:** Time schedule

Timepoint	Initial	Follow up			
	visit	1st month (± 7 days)	3rd month (± 14 days)	6th month (± 28 days)	12th month (± 28 days)
Eligibility screen	×				
Informed consent	×				
Baseline data					
Demographics	×				×
General medical history	×				
Physical examination	×	×	×	×	×
ECG	×		×		×
Echocardiography	×				×
Laboratory testing	×		×		×
Randomization	×				
CCTA (if assigned)	×				
Clinical procedure data					
Other CIT	×	×	×	×	×
CR	×	×	×	×	×
Complications	×	×	×	×	×
OMT	×		×		×
Radiation	×	×	×	×	×
HRQOL	×			×	×
Cardiovascular endpoints	×	×	×	×	×
Length of stay	×				

CIT: cardiac imaging testing; ECG: electrocardiogram; CR: coronary revascularization; OMT: optimal medication treatment; HRQOL: health-related quality of life

### Sample size

The sample size calculation is performed using R (version 3.6.4; R Foundation for Statistical Computing, Vienna, Austria). To compute a weighted average rate as the projected rate of CCTA without obstructive CAD for a diagnostic strategy, data from previous literatures used in the sample size calculation include rate of CCTA without obstructive CAD in high risk group and distribution of subjects in low and high risk group.

For NICE strategy, theses parameters fluctuated over a wide range in different cohorts [12, 13, 16]. Thus, the distribution of subjects and the rate of CCTA without obstructive CAD in high risk group is estimated to be approximately 2:3 for low/high risk group and 62%, respectively, resulting in a projected rate of 3/(2 + 3)×57%=37.2%. Similarly, we assume that the projected rate of primary endpoint is 2/(2 + 3)×57%=22.8% for ESC strategy, based on the results of the original study [11] as well as our previous studies including one which investigated the RF-CL model in subjects with borderline PTP [16, 17].

For the analysis of primary endpoint, on 2-sided test 5% significance level and for the 1:1 allocation ratio, a sample size of 800 (allowing for 10% noncompletion) will provide more than 99% power between NICE and ESC arm. Thus, the final sample size is chosen to be 800 subjects (400 in each randomization group).

### Statistical plan

All statistical analyses are performed by the statistical and data coordinating group using R (version 3.6.4; R Foundation for Statistical Computing, Vienna, Austria) or MedCalc (version 15.2.2; MedCalc Software, Mariakerke, Belgium). Two-tailed P < 0.05 is considered statistically significant. The primary analysis is conducted according to a modified intention-to-treat (ITT) principle. All subjects who undergone randomization and don’t withdraw before assigned intervention (CCTA or no further testing) or other CIT during the initial management will be included. Categorical variables will be described by counts and proportions and compared by chi-square tests or fisher exact test. Continuous variables will be described by mean and standard deviation or median and interquartile range and compared through t test or Mann-Whitney U tests.

The comparison of primary outcome between two diagnostic strategy regimens was expressed as risk ratio, along with 95% confidence interval and number needed to treat. The per-protocol analysis excluding subjects with insufficient compliance or with other major protocol deviation(s) and the traditional ITT analysis including all subjects who are randomized will be performed as prespecified sensitivity analyses. Different diagnostic metrics, such as initial CIT without positive result, unnecessary CCTA and CCTA with no sign of CAD, will also be considered as sensitivity analyses. To explore the consistency of strategy effectiveness according to specific subject characteristics, a limited number of prespecified subgroup analyses for the primary endpoint are conducted using interaction terms in log-binomial regression models. We will also undertake a hierarchical analysis (giving priority to clinical importance of the components of the composite outcome rather than time to first event: Death > nonfatal MI > hospitalization due to unstable angina > CCTA without obstructive CAD > referral to CIT) using the matched win ratio method [35].

Kaplan-Meier curves for cumulative event-free estimates survival from the first of the following: endpoints of concern, death, the end of 1-year follow-up period or loss to follow-up, are presented graphically and the Log-rank test will be used to calculate the corresponding p. A Cox proportional hazards model will be used to compare the time to study endpoint and to account for heterogeneity among subjects, the models will be adjusted for pre-specified and minimization covariates, which are selected on the basis of their established importance in other CCS cohorts, highly complete data capture, and a sufficient range of values for risk to vary among subjects meeting study eligibility criteria.

Multiple imputation using the R package Multivariate Imputation by Chained Equations algorithm will be used for missing data in ITT analysis of primary endpoint to ensure all subjects could be included in the ITT analysis, as well as HRQOL analysis [36]. Strategy comparisons of HRQOL are performed by using a mixed-effects regression model to account for repeated measures within a subject, with strategy, time point, baseline HRQOL and minimization covariates as predictor variables. If the analyses above show significant differences in study endpoints, information from the study and other sources, including medical costs, resource use, HRQOL and clinical outcomes will be used for the potential cost-effectiveness analyses to quantify the incremental cost per quality-adjusted life year gained.

## Discussion

Recent studies have demonstrated that performance of CIT was significantly influenced by PTP [37–39] and supported different diagnostic strategies to defer unnecessary CIT in patients at low risk [11, 14, 15]. However, only 32% of imaging centers in Europe selected optimal CIT based on estimation of PTP while 31% proceed directly to CCTA [40] and routine utilization of CCTA in low risk, even clinically healthy population were common in China [41]. Although current guidelines regarding the evaluation and management of SCP symptoms suggestive of CCS all emphasize the identification of patients unlikely to benefit from further CIT by structured and evidence-based diagnostic strategies [3–5], ample evidence of inappropriate referrals of CIT persists [6–9], which might be mainly attributed to the deficiency of consensus among clinicians and researchers on the preferred diagnostic strategy at the basis of specialized RCTs. Thus, the rationale and design outlined above are unique and desiderated in that the OPERATE trial will systematically compare two different diagnostic strategies in patients with SCP suggestive of CCS.

The risk of incorrectly deferral of CIT for patients with severe CAD should emerge as particularly strong candidates accounting for the overuse of CIT in clinical practice [1]. In fact, a risk of false negative will always be present for any testing and current guidelines recommended no further CIT in patients with low probability of obstructive CAD and cardiovascular events [3–5]. Thereby, identifying a proportion of patients at low risk to avoid CIT which may lead to false positive results and extra exposure of accompanying risk is acceptable, especially in an era of excess CIT use [42]. Data from more than 30, 000 CCS patients demonstrated that on the basis of contemporary OMT, most patients with angina were likely to experience resolution of symptoms without events or CR [43]. Moreover, both NICE and ESC strategy were externally validated in the CICM-SCP registry composed of patients from similar sites [16–18], which maximized the generalizabilities and reliabilities of them. Indeed, according to data from CICM-SCP registry composed of patients who underwent CCTA based on the decisions of local physicians, the proportion of patients who were classified into low risk group by NICE and ESC strategy but suffered MACE during a 17-month follow-up was only 1.1% and 0.7%, respectively [16]. On the other hand, the principle of OPERATE trial which left major decisions to local heart team members and referring physicians is intended to allow considerable leeway for clinical judgment in keeping with the pragmatic design. Also, this design in combination with ITT analysis can potentially elucidate the effectiveness of diagnostic strategies in routine clinical practice.

Although PROMISE minimal risk tool (PMRT) has been also validated in the CICM-SCP registry and demonstrated a robust risk assessment of SCP in several other validation studies [16, 44–46], OPERATE trial does not includes an arm of PMRT-based diagnostic strategy for the reasons below. First, the cutoff derivations of PMRT to identify low risk individual vary considerably among different cohorts [16, 44–47]. Second, instead of concerning about obstructive CAD, the PMRT defined minimal CAD risk as no coronary plaque or calcification and no MACE during follow-up. Third, the PMRT included a parameter from blood test, the high-density lipoprotein cholesterol level, as an independent variable. Except for ESC-PTP and RF-CL model, the coronary artery calcium score (CACS)-weighted clinical likelihood (CACS-CL) model which integrated CACS into estimation of PTP [11] was also recommended by the latest guideline for evaluation and diagnosis of chest pain [4] and externally validated in the CICM-SCP registry [16]. However, CACS scan is an extra test with radiation exposure and cost that potentially delays diagnosis and violates the hypothesis of identifying a subgroup for whom no further test is needed [18, 46]. As a result, a CACS-CL model-based diagnostic strategy has not been included by OPERATE trial so far.

Several representative RCTs [7, 9, 48, 49] and observational registry [16, 43] has provided obvious evidences that patients referred to CIT for suspected CCS had a very low rate of clinical event, resulting in a urgent demand to conservatively improve utilization of resources [1]. Meanwhile, the low event rate will limit our strength of evidence and causal statements inferred from an underpowered trial with a predefined primary outcome of MACE [1, 27]. To overcome this problem, we choose CCTA without obstructive CAD as primary endpoint to indicate effectiveness of the diagnostic strategy, which is, in turn, the main concerned outcome of PTP models, and introduce the approach of win ratio to analyze composite endpoint of MACE, CCTA without obstructive CAD and referral to CIT in the order of clinically importance. Similar designs using the imaging-based primary endpoints have also been selected by other comparative effectiveness trials of diagnostic strategies for SCP [20, 32, 50]. In addition, although it has been proposed that cardiac ischemia or plaque characteristics detected by CCTA to target intervention may improve clinical outcome, a detailed evaluation and treatment pathway have not been established [32, 51, 52]. Thus, anatomy severity remains the primary arbiter of post-CCTA management [4].

In patients with SCP suggestive of CCS, CCTA provided excellent performance for diagnoses and prognosis of CAD in a noninvasive and feasible way [27] and major guideline bodies have started to endorse incorporation of CCTA more definitively than before [3–5, 28]. Thus, we uniformly give CCTA the first-line status in subjects at high risk according to NICE and ESC strategy for several reasons. First, recent researches indicated that in terms of major clinical outcomes, CCTA-first strategy was superior or not inferior to NFT- or ICA-first strategy [7, 9, 49, 53, 54]. Second, CCTA are broadly available because of relatively low technical and personnel demands, while NFT, like positron emission tomography and stress cardiac magnetic resonance, although powerful, are much less available and their applicability is still limited by infrastructural and capacity requirements, especially in China [10]. Third, models constituting the ESC strategy were established in CCTA-based cohorts [3, 11]. Last, the post-CCTA clinical management paradigm based on CAD-RADS has been demonstrated to be associated with lower risk of MACEs and fewer invasive procedures [30].

In conclusion, we anticipate that the OPERATE study will investigate the real effects of symptom-focused (NICE) and PTP-based (ESC) diagnostic strategy on decisions of clinical management and subsequent clinical outcomes in patients with SCP suggestive of CCS.

### Electronic supplementary material

Below is the link to the electronic supplementary material.

Additional File 1: SPIRIT 2013 checklist

## Data Availability

Not applicable.

## Abbreviations

SCP: stable chest pain
CCS: chronic coronary syndrome
CAD: coronary artery disease
CCTA: coronary computed tomography angiography
CIT: cardiac imaging testing
NICE strategy: 2016 U.K. National Institute of Health and Care Excellence guideline-determined diagnostic strategy
ESC strategy: 2019 European Society of Cardiology guideline-determined diagnostic strategy
PTP: pretest probability
RCT: randomized controlled trial
MACE: major adverse cardiac events
ECG: electrocardiogram
NFT: noninvasive functional testing
ICA: invasive coronary angiography
OMT: optimal medication treatment
PMRT: PROMISE minimal risk tool
ITT: intention-to-treat
HRQOL: health-related quality of life
CACS-CL: coronary artery calcium score-weighted clinical likelihood
RF-CL: risk factor-weighted clinical likelihood
CR: coronary revascularization
